# Anatomy and Connectivity of the Torus Longitudinalis of the Adult Zebrafish

**DOI:** 10.3389/fncir.2020.00008

**Published:** 2020-03-13

**Authors:** Mónica Folgueira, Selva Riva-Mendoza, Noelia Ferreño-Galmán, Antonio Castro, Isaac H. Bianco, Ramón Anadón, Julián Yáñez

**Affiliations:** ^1^Department of Biology, Faculty of Sciences, University of A Coruña, Coruña, Spain; ^2^Centro de Investigaciones Científicas Avanzadas (CICA), University of A Coruña, Coruña, Spain; ^3^Department of Neuroscience, Physiology and Pharmacology, University College London, London, United Kingdom; ^4^Department of Functional Biology, Faculty of Biology, University of Santiago de Compostela, Santiago de Compostela, Spain

**Keywords:** optic tectum, pretectum, cerebellum, electron microscopy, neural tracing, connections

## Abstract

This study describes the cytoarchitecture of the torus longitudinalis (TL) in adult zebrafish by using light and electron microscopy, as well as its main connections as revealed by DiI tract tracing. In addition, by using high resolution confocal imaging followed by digital tracing, we describe the morphology of tectal pyramidal cells (type I cells) that are GFP positive in the transgenic line *Tg(1.4dlx5a-dlx6a:GFP)*^ot1^. The TL consists of numerous small and medium-sized neurons located in a longitudinal eminence attached to the medial optic tectum. A small proportion of these neurons are GABAergic. The neuropil shows three types of synaptic terminals and numerous dendrites. Tracing experiments revealed that the main efference of the TL is formed of parallel-like fibers that course within the marginal layer of the optic tectum. A toral projection to the thalamic nucleus rostrolateralis is also observed. Afferents to the TL come from visual and cerebellum-related nuclei in the pretectum, namely the central, intercalated and the paracommissural pretectal nuclei, as well as from the subvalvular nucleus in the isthmus. Additional afferents to the TL may come from the cerebellum but their origins could not be confirmed. The tectal afferent projection to the TL originates from cells similar to the type X cells described in other cyprinids. Tectal pyramidal neurons show round or piriform cell bodies, with spiny apical dendritic trees in the marginal layer. This anatomical study provides a basis for future functional and developmental studies focused on this cerebellum-like circuit in zebrafish.

## Introduction

The torus longitudinalis (TL) is a paired elongated neural structure lying along the medial margins of the optic tectum, suspended from the intertectal commissure and protruding into the tectal ventricle (Ito, 1971; Wullimann, 1994; Northmore, 2011). Discovered by Carus in 1814, the TL was first thought considered to be homologous to the mammalian fornix until Rabl-Rückhard (1884) showed that the TL is a structure exclusive to fish, in particular, the ray-finned fishes: chondrosteans, holosteans and teleosts (for a review of the early history of the TL, see Sargent, 1903). An ultrastructural study in the carp TL described three neuron types and synaptic glomeruli in the neuropil (Ito, 1971). In addition, a few studies using immunohistochemistry (Kageyama and Meyer, 1989; Folgueira et al., 2007) or *in situ* hybridization (Maruska et al., 2017), have revealed that glutamatergic neurons are much more numerous than GABAergic cells in the teleost TL.

The TL is closely associated with the optic tectum both in terms of its anatomical location and its connectivity (Sala, 1895; Kudo, 1923). Golgi studies reported axonal projections from TL granule cells to the most superficial layer of the tectum, the marginal layer or stratum marginale (Sala, 1895; Sajovic and Levinthal, 1982). This TL efferent connection was then traced experimentally to the optic tectum in the holostean longnose gar (*Lepisosteus osseus*; Northcutt and Butler, 1980) and several teleosts (Grover and Sharma, 1981; Luiten, 1981; Northcutt, 1982; Wullimann and Northcutt, 1990; Ito et al., 2003; Xue et al., 2003; Folgueira et al., 2007; Zeymer et al., 2018). Granule cell axons run in the marginal layer parallel to the surface of the tectum, in a manner that is similar to cerebellar parallel fibers, and synapse onto the spiny, branched dendrites of pyramidal or type I cells (Laufer and Vanegas, 1974; Vanegas et al., 1974). A correlation between the size of the tectum or the thickness of the marginal layer and the size of the TL has been noted in different teleosts (Kudo, 1923; Kishida, 1979). Connectivity between tectum and TL is reciprocal with tectal type X cells making efferent projections into TL (Meek and Schellart, 1978; Ito et al., 2003; Xue et al., 2003; Folgueira et al., 2007).

The toro-tectal circuit involving the marginal layer and pyramidal cells is considered a “cerebellum- like” circuit because of its resemblance to the “parallel fiber- Purkinje cell” circuit in the cerebellum (Bell, 2002; Bell et al., 2008). Other cerebellum-like systems include the medial and dorsal octavolateralis nucleus (present in some aquatic vertebrates), the electrosensory lobe (present in some bony fish), the rostrolateral nucleus (present in a few bony fish), and the dorsal cochlear nucleus (present in most mammals) (see Bell, 2002 and Bell et al., 2008 for reviews on cerebellum-like structures). Why and how such systems appeared during evolution are still open questions (Bell, 2002; Bell et al., 2008; Northmore, 2017).

Experimental studies of the connections of the TL in teleosts using tracing methods have revealed that the TL receives afferents mainly from the pretectal nuclei, the subvalvular nucleus and the cerebellum (carp: Ito and Kishida, 1978; Ito and Yoshimoto, 1990; Ito et al., 2003; holocentrids: Xue et al., 2003; trout: Folgueira et al., 2007).

The close anatomical relationship between TL and the optic tectum led early authors to suggest that the TL may play a part in both gravistatic and photostatic functions of the midbrain (Kudo, 1923; Ohta, 1959). More recently, electrophysiological recordings in the TL of carp and percomorphs showed two types of units: (1) photic, that respond with sustained discharges to dimming light in the contralateral visual field, and (2) saccadic, that respond with bursting discharges synchronized with saccadic eye movements (Northmore et al., 1983; Northmore, 1984). Photic responses in TL appear to be achromatic, unlike responses in the optic tectum (Gibbs and Northmore, 1998). These results showing photic and saccadic units (Northmore et al., 1983; Northmore, 1984) also provided physiological evidence of reciprocal topographic connections between the TL and the optic tectum. Moreover, electrophysiological studies confirmed the existence of a precisely ordered topographical loop between dorsomedial TL and optic tectum. This topographical mapping over-represents the visual horizon, and is transmitted with some delay from the TL via the marginal fibers over the tectum (Northmore, 2011). Based on the photometric response, it had been suggested that the TL may play a role in the dorsal light reflex, which orients the back of the fish body toward the brighter light source (Gibbs and Northmore, 1996). More recently, based on network modeling, Northmore (2017) proposed that the TL-optic tectum system is involved directing selective visual attention and maintaining attention on a visual target between saccadic eye movements.

Little is known about the anatomy and connectivity of the TL in the adult zebrafish, except for its general morphology (Wullimann et al., 1996), its projections to the stratum marginale (Sajovic and Levinthal, 1982) and its connections with pretectal areas (Yáñez et al., 2018). Larval zebrafish are nowadays a prime vertebrate model to dissect the anatomy and function of neural circuits using neurogenetic and optogenetic methods (Wyatt et al., 2015; White, 2016; Förster et al., 2017; Robles, 2017; Vanwalleghem et al., 2018). Although a number of studies have analyzed the anatomical organization, cell types, connectivity and function of the optic tectum in zebrafish larvae (Del Bene et al., 2010; Nevin et al., 2010; Robles et al., 2011, 2013, 2014; Corbo et al., 2012; Yokogawa et al., 2012; Heap et al., 2013, 2018a,b; Portugues et al., 2014; Temizer et al., 2015; Bianco and Engert, 2015; Dunn et al., 2016; Filosa et al., 2016; Thompson and Scott, 2016; Thompson et al., 2016; Helmbrecht et al., 2018; Kunst et al., 2019), little is known about TL. To our knowledge, only two studies have described aspects of the toro-tectal circuit in zebrafish larva. One describes tectal cell types (including type X cells) based on transgene expression (DeMarco et al., 2019), while the other reports neural responses in the TL as a result of visual stimulation (Portugues et al., 2014).

Our study describes the cellular organization of the adult zebrafish TL using light and electron microscopy, and analyses its main connections using carbocyanine tract tracing. We also analyze the transgenic line *Tg(1.4dlx5a-dlx6a:GFP)*^*ot*1^ in which pyramidal tectal cells are labeled. Thus, this work on the zebrafish TL and related structures opens the door for future structural, developmental and functional studies of this very intriguing cerebellum-like system both in the adult and larva.

## Materials and Methods

### Animals

Thirty-two wild type zebrafish (*Danio rerio*) were used in this study for neural tracing, immunohistochemistry and electron microscopy. In addition, one individual of *Tg(gfap:GFP)*^*mi*2001^ and seven individuals of *Tg(1.4dlx5a-dlx6a:GFP)^ot^* [two at 20 days post-fertilization (dpf) and five adults] were also used. Prior to all experiments, animals were euthanized by methanesulfonate salt (MS222; Sigma-Aldrich, St Louis, MO, United States) overdose. Animal handling and experimental procedures conformed to European Community’s guidelines on animal care and experimentation and were approved by the UCL Animal Welfare Ethical Review Body and the United Kingdom Home Office under the Animal (Scientific Procedures) Act 1986.

### Light and Electron Microscopy

For light and transmission electron microscopy, two adult zebrafish were fixed by intracardial perfusion with cold 2% paraformaldehyde and 1% glutaraldehyde in 0.1 M phosphate buffer pH 7.4 (PB), and heads were kept in the same fixative for 12 h at 4°C. Brains were then removed, washed and kept in PB at 4°C. Postfixation was made with 1% osmium tetroxide in PB for 2 h, and then brains were rinsed, dehydrated and embedded in Spurr’s resin. Sectioning was made using an ultramicrotome (Ultracut E 701704, Leica AG Reichert). Transverse semithin sections (1 μm thick) through the rostral and intermediate region of the TL were collected on slides, stained with toluidine blue-borax and analyzed using light microscopy. Ultrathin sections (70-80 nm thick) were collected on formvar carbon-coated grids, stained sequentially with lead citrate and uranyl acetate and observed and photographed in a transmission electron microscope (JEM 1010, JEOL) equipped with a digital camera (Olympus).

In addition, we used Nissl and hematoxylin-eosin stained series of transverse and longitudinal sections of the adult zebrafish brain from our collections.

### Immunohistochemistry

For immunohistochemistry against glutamic acid decarboxylase (GAD), we used series of transverse sections of two adult brains immunostained with a primary antibody against GAD67 (Chemicon, Temecula, CA, United States, dilution 1:1000; Code AB108). The protocols and controls for GAD immunohistochemistry in the zebrafish brain were as published elsewhere (Castro et al., 2006; Folgueira et al., 2007). Briefly, zebrafish were fixed by transcardial perfusion with 4% paraformaldehyde. Their brains were cryoprotected in 30% sucrose in PB, frozen with liquid nitrogen, and cut on a cryostat (12 μm thick). Sections were mounted on gelatinized slides, rinsed in PB saline (PBS) and incubated with normal goat serum (Sigma, 1:100) and then with the primary GAD67 antibody overnight. The next day, sections were washed in PBS, incubated with secondary antibody goat anti−rabbit (Sigma; 1:100) for 1 h, washed in PBS, and incubated in rabbit PAP complex (Sigma, 1:400) for 1 h. The immunoreaction was developed with 0.005% diaminobenzidine (DAB; Sigma) and 0.003% H_2_O_2_.

Immunofluorescence against green fluorescent protein (GFP) in *Tg(gfap:GFP)*^*mi*2001^ and synaptic vesicles 2 (SV2) in wild-type fish was performed following a similar protocol to the described for GAD67 staining. In this case, cryosections were incubated first with a primary antibody for one hour and then with a fluorescent secondary antibody. Primary and secondary antibodies and dilutions were: rabbit anti-GFP (Torrey Pines Biolabs; dilution 1:1000, TP401), mouse anti-synaptic vesicle protein 2 (DSHB, dilution 1:250, AB 2315387), goat anti-rabbit Alexa Fluor 488 (Invitrogen, dilution 1:500, A-11034), goat anti-mouse Alexa Fluor 568 (Invitrogen, dilution 1:500, A-11031). In the case of SV2 immunofluorescence, sections were counterstained using SYTOX^TM^ Blue Nucleic Acid Stain (ThermoFisher, dilution 1:1000, S11348).

For immunofluorescence against GFP in *Tg(1.4dlx5a-dlx6a:GFP)*^*ot*1^, after fish euthanasia, brains were fixed in 4% paraformaldehyde for at least 24 h and then transferred to PB saline (PBS). For adult brains, they were embedded in 3% agarose, sectioned (transverse sections, 50-100 μm thick) using a vibratome (Vibroslice, Campden Instruments, United Kingdom) and collected in small volume tubes. After washing sections in PBS with 0.5% Triton X-100 (PBST), they were incubated with normal goat serum in PBST (Sigma; 1:1000) for 1 h and next with a primary antibody (rabbit anti-GFP; Torrey Pines Biolabs, dilution 1:1000, TP401) for 24 h. The next day, sections were washed in PBST and incubated with secondary antibody (goat anti rabbit- Alexa Fluor 488, Invitrogen dilution 1:500, A-11034) for 1 h. After washing the sections in PBST, they were transferred onto gelatin-coated slices using 50% glycerol in 0.1 M PB as mounting media. For whole mount immunofluorescence in 20 dpf fish, brains were transferred to 100% methanol and maintained at −20°C for 1 h. Next they were transferred to 50% methanol and PBS and permeabilized using proteinase K (Sigma; 1:1000, 30 min). After rinsing and fixation in 4% paraformaldehyde (40 min), they were then incubated with normal goat serum for 1 h and with the primary antibody overnight. The next day, after washes in PBST, brains were and incubated with secondary antibody overnight. After washes in PBST, brains were then mounted for confocal imaging in 1.5% agarose in PB.

### Neuronal Tracing

For neuronal tracing from the TL, twelve individuals were deeply anesthetized with MS222, perfused transcardially with 4% cold paraformaldehyde in PB and kept in the same fixative until use. For carbocyanine labeling experiments, each brain was embedded in a block of 3% agarose and sectioned on a vibratome from a rostral or caudal approach until reaching the intermediate (6 cases) or caudal (4 cases) region of the TL. DiI labeling of the rostral TL was ruled out, as these experiments would probably result in unspecific labeling of systems using the posterior commissure. A minute crystal of the carbocyanine dye DiI (1,1′-dioctadecyl-3,3,3′3′-tetramethylindocarbocyanine perchlorate; Invitrogen, Eugene, OR, United States) was inserted in the exposed TL by using a minute insect pin (000). The surface was sealed with melted agarose and the block was incubated in fixative for two weeks in darkness at 37°C. Additionally, two specimens were used to label the TL in whole- mount, inserting dorsally a small DiI crystal. In one case, to minimize diffusion of the tracer along tracts including the intertectal commissure, two longitudinal cuts were made along the dorsal optic tectum. The injection site was sealed and the brain was embedded in agarose and incubated as before. After incubation, brain blocks were sectioned transversally on a vibratome and mounted on slides in 50% glycerol in 0.1 M PB.

DiI is a lipophilic tracer that diffuses passively along the cell membrane, labeling the whole neuron from the application point. Therefore, complementary reciprocal experiments were necessary to confirm the connections of TL. Tracer applications to putative torofugal or toropetal brain areas were made in seventeen fixed brains. The areas labeled in whole brains were the optic tectum (3 cases), the cerebellar corpus (3 cases), the cerebellar granular eminence (1 case) and the cerebellar valvula (3 cases, after lateral ablation of an optic lobe). DiI was also applied in sectioned brains to various nuclei: the nucleus rostrolateralis (1 case), the nucleus isthmi (3 cases), the accessory and central pretectal nuclei (APN/PCe, 1 case) and the paracommissural pretectal nucleus (PCo) (2 cases) (see also Yáñez et al., 2018). For clarity, only observations related to TL will be described for these complementary experiments.

### Fluorescence Imaging

Fluorescent sections were photographed in an epifluorescence microscope (Eclipse 90i; Nikon) equipped with a digital camera (DP70; Olympus) using appropriate fluorescence filters. Selected sections of DiI labeling and *Tg(1.4dlx5a-dlx6a:GFP)*^*ot*1^ fish were also imaged using a laser scanning confocal microscope (A1R, Nikon) and confocal stacks were analyzed using ImageJ software (Fiji). For *Tg(1.4dlx5a-dlx6a:GFP)*^*ot*1^, a total number of 13 different pyramidal cells were imaged using a Nikon Plan Fluor 20x/0.50 objective with a 0.2-0.3 μm *z*-step between optical sections. Pyramidal cells were reconstructed from volumetric data using Simple Neurite Tracer plugin in Fiji. Maximal projections of rendered paths (neurites) generated in Fiji were vectorized using Corel Draw^®^ (Ottawa, ON, Canada).

For presentation in plates, most fluorescence digital images were converted to gray scale, inverted such that labeled cells and fibers appear black on a white background, and adjusted for brightness and contrast with Corel Suite^®^ (Ottawa, ON, Canada). The nomenclature used for brain regions follows that of Wullimann et al. (1996) and Yáñez et al. (2018).

## Results

### General Morphology and Cellular Organization of the Zebrafish Torus Longitudinalis

**FIGURE 1:**
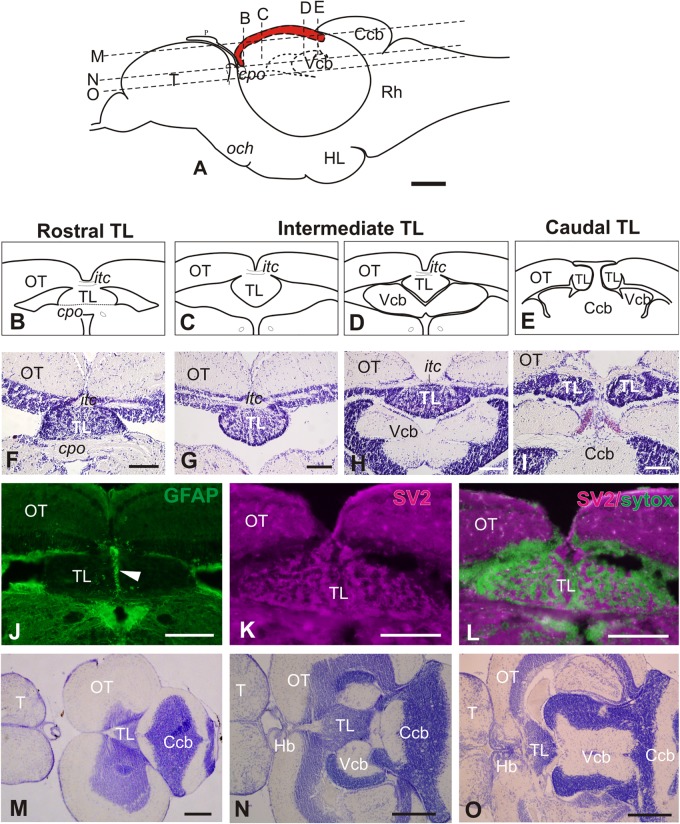
General morphology of the adult zebrafish Torus Longitudinalis (TL). **(A)** Schema of the brain in a lateral view representing the location of the TL (in red) along the rostro-caudal axis. Anterior to the left. The approximate levels of sections **(B–D)** and **(K–M)** are also shown. **(B–E)** Diagrams of the general anatomy of the TL at rostral, intermediate and caudal levels in transverse section. **(F–I)** Images of Nissl stained sections showing TL at approximately the same rostrocaudal levels as **(B–E)**. **(J)** Glial processes along TL midline (arrowhead) after GFP immunostaining in *Tg(gfap:GFP)*^*mi*2001^. **(K)** Neuropil in TL showing immunoreactivity against synaptic vesicles 2 (SV2). **(L)** Merged channels for SV2 (magenta) and Sytox green (green). Note that the non-immunoreactive areas in **(I)** correspond with location of cell bodies in green). **(M–O)** Horizontal sections [at levels indicated in **(A)**] showing the general anatomy and location of TL. Anterior to the left. For abbreviations, see list. Scale bars: 100 μm **(A-L)**, 250 μm **(M-O)**.

**FIGURE 2:**
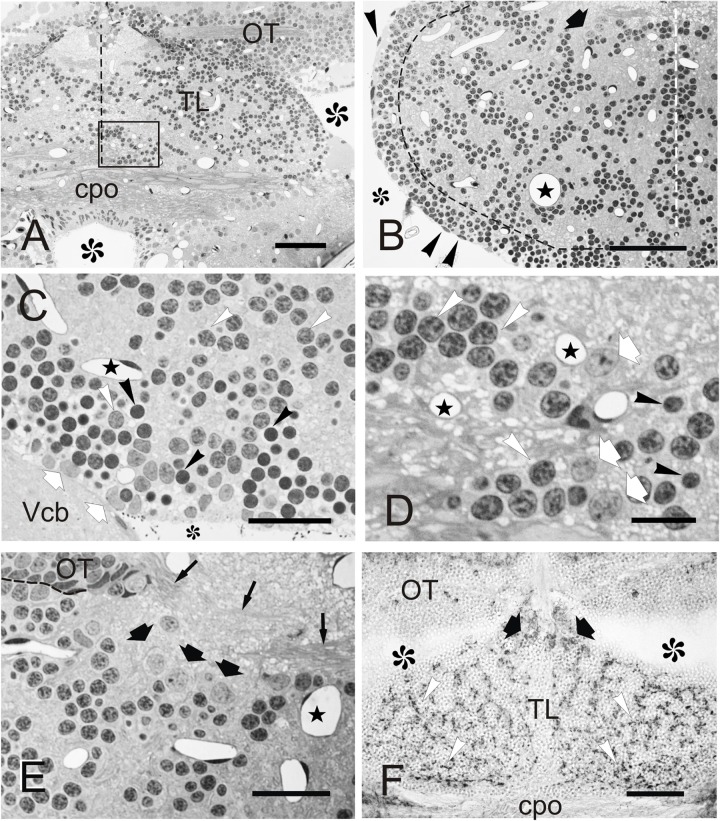
Cellular organization of zebrafish TL. **(A–F)** Micrographs of semithin transverse sections of zebrafish TL stained with toluidine blue-borax **(A–E)** and cryostat transverse sections immunostained against GAD67 **(F)**. **(A)** Section through the rostral TL. Note that at this level, TL sits on top of the posterior commissure. Midline is indicated by dashed line. Area squared is shown in detail in **(D)**. **(B)** Section through the intermediate region of the TL showing the peripheral band of cells and internal cell clusters. Black dashed line marks the approximate limit between both populations. Note also the presence of a group of large neurons (black thick arrow) in dorsal TL and the nuclei of ependymal cells (arrowheads). White dashed line marks the midline. **(C)** Detail of the peripheral band of cells below the ventricular surface showing small (**Nu_2_**, black arrowheads) and medium size (**Nu_1_**, white arrowheads) round nuclei, as well as more irregular nuclei with paler chromatin (**Nu_3_**, white arrows). Midline is to the right. **(D)** Detail of the area squared in **(A)** showing small (**Nu_2_**, black arrowheads) and medium size nuclei (**Nu_1_**, white arrowheads), as well as slightly larger nuclei (**Nu_3_**, arrows) with paler chromatin. Dashed line marks the OT border. **(E)** Section showing the location of large **(C_3_)** cells in the dorsal region of TL (thick black arrows). Thin arrows: axons of the intertectal commissure. Midline is to the right. **(F)** Section through rostral TL showing GAD67-positive cell bodies (thick black arrows) in dorsal areas, as well as immunopositive fibers (white arrowheads). Asterisks, ventricle; Stars, blood vessels. For abbreviations, see the list. Scale bars: 50 μm **(A–B, F)**; 20 μm **(C,E)**; 10 μm **(D)**.

In the adult zebrafish, the torus longitudinalis (TL) is a paired elongated body attached below the median side of the optic lobes below the intertectal commissure and protruding into the tectal ventricle (Figures 1A–I). The adult TL is about 1mm long and can be roughly subdivided into three regions based on morphology and location along the rostro-caudal axis: rostral, intermediate and caudal (Figures 1A–E). The rostral TL sits on top of the posterior commissure with its two halves fused forming a slightly flattened structure (Figures 1B,F, 2A). The intermediate TL is longer than the other two, with both halves still fused at the midline and measuring about 250 μm high and 350 μm wide. Rostrally, intermediate TL hangs into the tectal ventricle (Figures 1C,F), while a bit more caudally it is flanked by the cerebellar valvula laterally and/or ventrally (Figures 1G,H). GFP expression in *Tg(gfap:GFP)*^*mi*2001^ shows a thick bundle of radial glial processes running along the TL midline both at rostral (Figure 1J) and intermediate levels (not shown). Expression of the tight junction protein zonula occludens 1 (ZO1) in the zebrafish larva (not shown) indicates that the apical surface of TL faces toward the tectal ventricle. The caudal TL sits dorsal or lateral to the cerebellar corpus, with both halves now separated into two lobes (Figures 1E,I). After staining against synaptic vesicle 2 (SV2), we observed intense immunoreactivity in the neuropil areas (Figures 1K,L) that are distributed among cell clusters in TL. In horizontal sections (Figures 1A,M–O), from dorsal to ventral, TL is first observed as a triangular structure located just anterior to the cerebellar corpus (Figures 1A,M). Then, more ventrally, TL acquires an elongated shape and its flanked by the cerebellar valvula (Figures 1A,N). At most ventral horizontal planes, TL shows a triangular shape and is located caudal to the habenulae and rostral to the cerebellar valvula (Figures 1A,O).

In semithin transverse sections, zebrafish TL is composed mainly of a number of small to medium-sized neurons and neuropils, as well as abundant capillaries (Figures 2A–E). At the ventricular surface of the TL, we could distinguish cells with flattened dark nuclei and cells with round and oval pale nuclei (Figures 2B,C). Beyond the ventricular surface, most toral cells showed round nuclei surrounded by little cytoplasm (Figures 2A,B). These cells are distributed in a peripheral band of densely packed cells and in internal cords or clusters interspersed by neuropil areas (Figures 2A,B). In both regions, cells are either medium size with clear stained nucleus (**C_1_**) or small with densely stained nucleus (**C_2_**), the latter being far more abundant in the peripheral band (Figure 2C). Because of their abundance and characteristics, these cells (**C_1_** and **C_2_**) are most likely granule cells. Many fewer cells have a larger pale nucleus surrounded by more abundant cytoplasm and with one nucleolus (**C_3_**). This cell type was mainly located at dorsal areas of TL, close to the intertectal commissure and in ventrolateral areas close to the ventricular surface (Figures 2B,D,E). The nuclei of **C_1_-C_3_** cells are further described based on ultra-thin sections (see Section “Fine Structure of the Adult Zebrafish Torus Longitudinalis Analyzed by Electron Microscopy” below). Larger (**C_3_**) cells could correspond to a GABAergic population, as described in trout (Folgueira et al., 2007). By performing an immunostaining against glutamic acid decarboxylase (GAD67), we observed that while most TL cell bodies were GAD-negative, a few were slightly larger and faintly GAD-positive, being located mainly dorsally in the TL, as is the case for **C_3_** cells. In addition, we observed abundant GAD-positive fibers evenly distributed throughout TL neuropil (Figure 2F). These GAD-positive fibers could originate from TL GAD-positive cell bodies, which could represent local interneurons as described in trout (Folgueira et al., 2007).

### Fine Structure of the Adult Zebrafish Torus Longitudinalis Analyzed by Electron Microscopy

**FIGURE 3:**
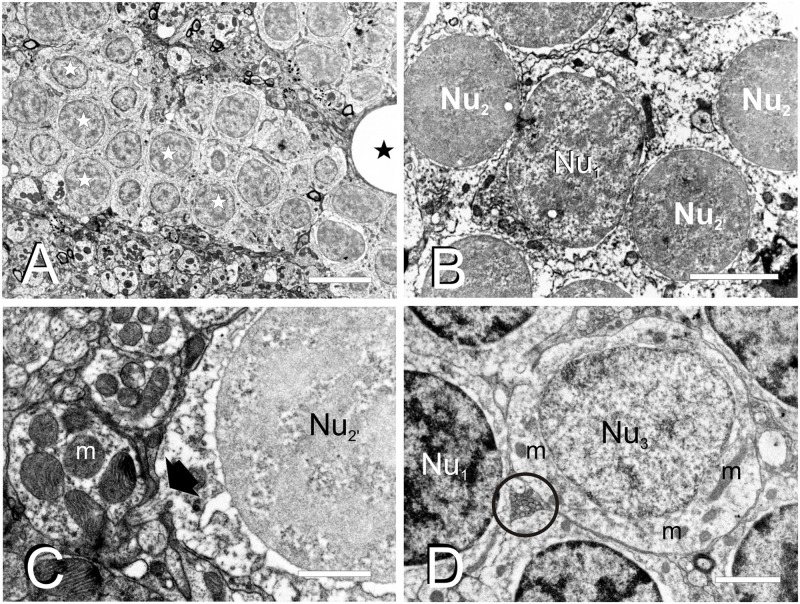
Fine cell structure of the adult TL. **(A–D)** Electron micrographs showing the main three nucleus types found in TL. **(A)** Low magnification electron micrograph showing a cell cluster with medium-sized round nuclei with partially condensed chromatin (**Nu_1_**, white star) and surrounded by a dense neuropil. **(B)** Detail of a medium-sized nucleus **(Nu_1_)** surrounded by smaller nuclei (**Nu_2_** and **Nu_2__’_**). **(C)** Detail of a small-sized cell nucleus with non-homogeneously condensed chromatin **(Nu_2__’_)** showing the exit of the axon (black arrow) from the cell body. **(D)** Nucleus **(Nu_3_)** with pale chromatin that belongs to the large-sized cells among other nuclei with more condensed chromatin **(Nu_1_)**. Note also the small compact bundle of non-myelinated axons (circle). m, mitochondria; black stars, blood vessels. Scale bars: 5 μm **(A)**, 2 μm **(B,D)**, 1 μm **(C)**.

By studying ultrathin sections, we could further characterize the nuclei of the three main cell types (**C_1_**, **C_2_**, and **C_3_**) identified in semithin sections of TL (Figure 3). The most abundant nuclei (**Nu_1_**)**** belong to **C_1_** cells, distributed in clusters throughout TL. They are medium-sized (5.2 ± 0.9 μm; *n* = 20) and round, with partially condensed chromatin (Figures 3A,B). The second nucleus type (**Nu_2_**)**** is smaller (3.3 ± 0.4 μm; *n* = 20), dark and round and displayed generally smooth chromatin that was evenly distributed, although differences in chromatin condensation can be noticed (**Nu_2_** and **Nu_2__’_**, Figures 3B,C). These nuclei belong to **C_2_** cells that are mainly located at the ventrolateral periphery of the intermediate TL, intermingled with **Nu_1_** nuclei. As **Nu_1_** and **Nu_2_** are the most abundant nucleus types, they probably belong to granule cells. A third, less frequent, nucleus type (**Nu_3_**) is mainly located in dorsal and ventrolateral regions of TL (Figure 3D). They are medium-sized (5.5 ± 1.5 μm, *n* = 7), with paler sparsely condensed chromatin and a nucleolus. These nuclei were mostly round, but some also appeared slightly flattened or even irregular (Figure 3D). Occasionally, they acquire a lobed shape because of an invagination in their nuclear envelope. These nuclei belong to larger cells (**C_3_**) that could be GABAergic interneurons, as stated earlier.

**FIGURE 4:**
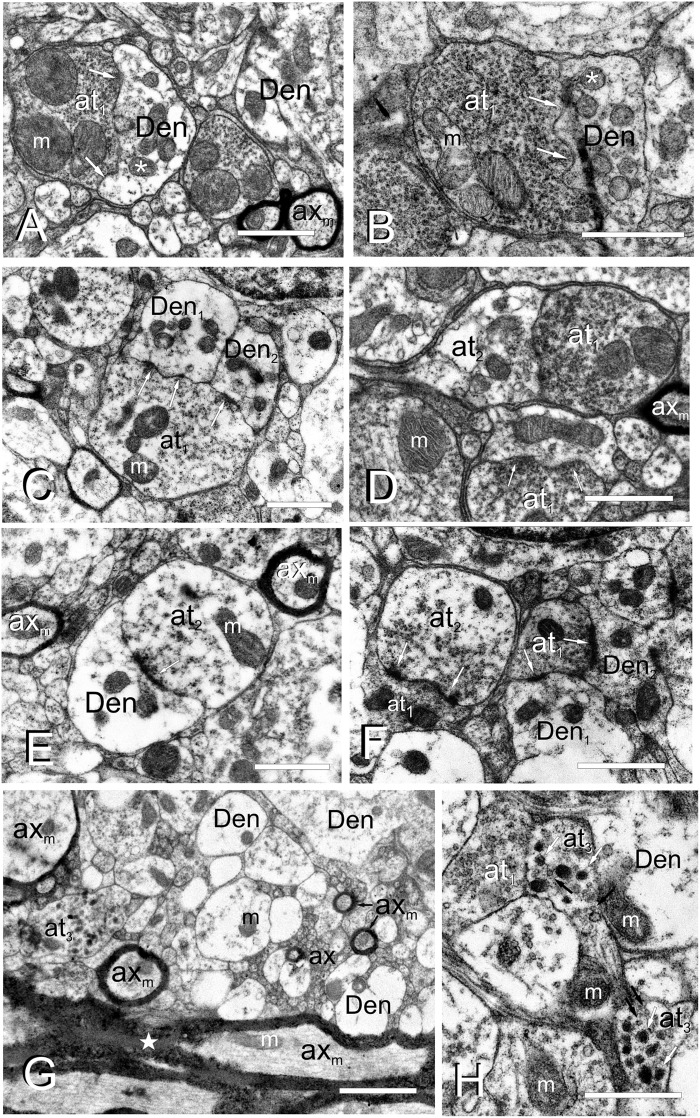
Fine cell processes structure of the adult TL. **(A–H)**, Electron micrographs of the TL neuropil showing different axon terminals and dendrites. **(A)** Type I axon terminal **(at_1_)**, with a number of small vesicles and large mitochondria (m) besides a dendritic process **(Den)** with smaller mitochondria (asterisk). Arrows: wavy-shaped synaptic active zone. **(B)** Type I axon terminal **(at_1_)** densely filled with vesicles in close contact with a dendrite **(Den)**. Arrows: wavy-shaped synaptic active zone. Note that mitochondria in the post-synaptic dendrite (asterisk) are smaller and more abundant. **(C)** Axon terminal type I **(at_1_)** making synapse (arrows) with two dendritic processes **(Den1**, **Den2)**. **(D)** Axo-axonic contact between type I (at_1_) and type II (at_2_) terminals, as well as a type I axo-dendritic synapse (arrows). **(E)** An axo-dendritic synapse (arrow) for a type II axon terminal. Note a myelinated axons **(ax_*m*_)** on the top-right and left. **(F)** Axo-axonic synapse (arrows) between type I **(at_1_)** and II **(at_2_)** terminals, as well as axo-dendritic synapses (arrows) with two dendritic processes **(Den_1_**, **Den_2_)**. **(G)** Axon terminal type III **(at_3_)** in the rostral TL intermingled with myelinated (ax_*m*_) and unmyelinated **(ax)** axons and dendrites **(Den)**. Note the thick and highly myelinated axons in the posterior commissure at the bottom of the image (star). **(H)** Detail of axon terminals type III **(at_3_)**, with small clear (black arrows) and large electron-dense vesicles (white arrows), next to type I **(at_1_)** axon terminals. m, mitochondria. Scale bars: 1 μm **(A–F,H)**; 2 μm **(G)**.

Torus Longitudinalis neuropil contains a number of cell processes with different diameters and characteristics, mainly thin dendrites and very thin unmyelinated axons, but also some myelinated axons (Figures 4A–H). Although the exit of a process from its parent cell is generally difficult to find, occasionally we did observe a thin process (0.2-0.3 μm in diameter) that contained a few microtubules exiting from the cell surface (Figure 3C). In the neuropil, dendrites have a pale appearance, lack synaptic vesicles and show scarce mitochondria. These dendrites appear to form *en passant* or terminal dilatations of about 1 μm in diameter that are postsynaptic to axon terminals, forming together axo-dendritic synapses (Figures 4A–F). Owing to their abundance, most of these dendritic dilatations probably come from granule cells. No dendritic spines or unusual specializations were observed in the dendrites of toral neurons. We observed compact groups of very thin unmyelinated axons (about 0.1 μm in diameter) located among cell bodies in the neuropil (**ax**, Figures 3D, 4G). These axons are very abundant throughout TL, so they probably originate from granule cells. In addition, we also observed thin myelinated axons (**ax**_*m*_, 0.5-1.5 μm) of unknown origin (probably toral afferent fibers) that run longitudinally in the neuropil (Figures 4A,D,E,G).

Three types of axon terminals (**at**) were distinguished based on their electron density, which appears to be correlated with the abundance, size and appearance/content of their synaptic vesicles. Type I axon terminals (**at**_1_) contain a number of small (30-40 nm) electron lucent synaptic vesicles that fill most of the terminal (Figures 4A–D,F,H). They usually contain 1-3 mitochondria with laminar cristae (Figures 4A–D) that are larger and more abundant than the mitochondria in the post-synaptic dendrites. These axon terminals normally synapse onto 1-2 dendrites through one or two wavy-shaped synaptic active zones (Figures 4A–C). Type II axon terminals (**at**_2_) appear the same size and shape as type I, but they have fewer synaptic vesicles next to the synaptic active zone (Figures 4D–F). These terminals also showed 1 or 2 mitochondria, which are again larger and less numerous than those in the postsynaptic dendrite (Figures 4D–F). A few axon dilatations (type III axon terminals, **at**_3_) were also seen in the rostral TL close to the posterior commissure (Figures 4G,H), although they did not appear to form typical synaptic contacts. These terminals showed large dense-core vesicles (100-120 nm in diameter) and a few small clear vesicles (50 nm) (Figure 4H). No mitochondria could be observed in the very few type III terminals we found. The origin of these three types of terminals was not assessed.

### Connections of the Adult Zebrafish TL Visualized by Direct DiI Application

**FIGURE 5:**
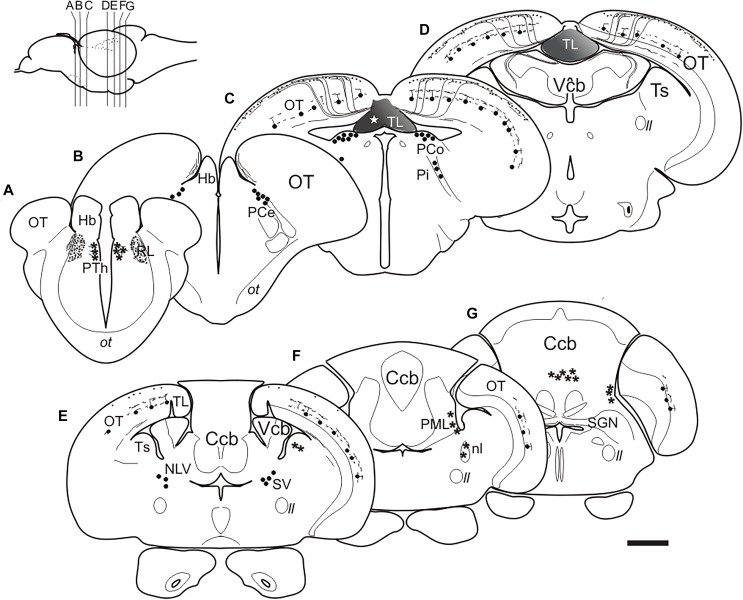
**(A–G)**, Schematic drawings of selected transverse sections of the brain of adult zebrafish from rostral to caudal at the levels indicated in the inset, showing the results of DiI application to the intermediate TL (star). Circles, cells labeled consistently; asterisks: cells labeled occasionally; lines and dots, labeled fibers. Dashed lines: areas of the brain. For abbreviations see the list. Scale bar for sections: 200 μm.

Tracer application to the TL leads to consistent labeling of characteristic fibers and cells in the optic tectum (toropetal tectal cells), as well as cells in three pretectal nuclei and in an area located ventrolaterally to the nucleus lateralis valvulae (Figure 5). Occasionally, we also observed labeled structures in other areas of the brain, including the nucleus rostrolateralis, prethalamic nucleus, torus semicircularis, nucleus isthmi, cerebellar corpus and posterior mesencephalic lamina (Figures 5A–G).

**FIGURE 6:**
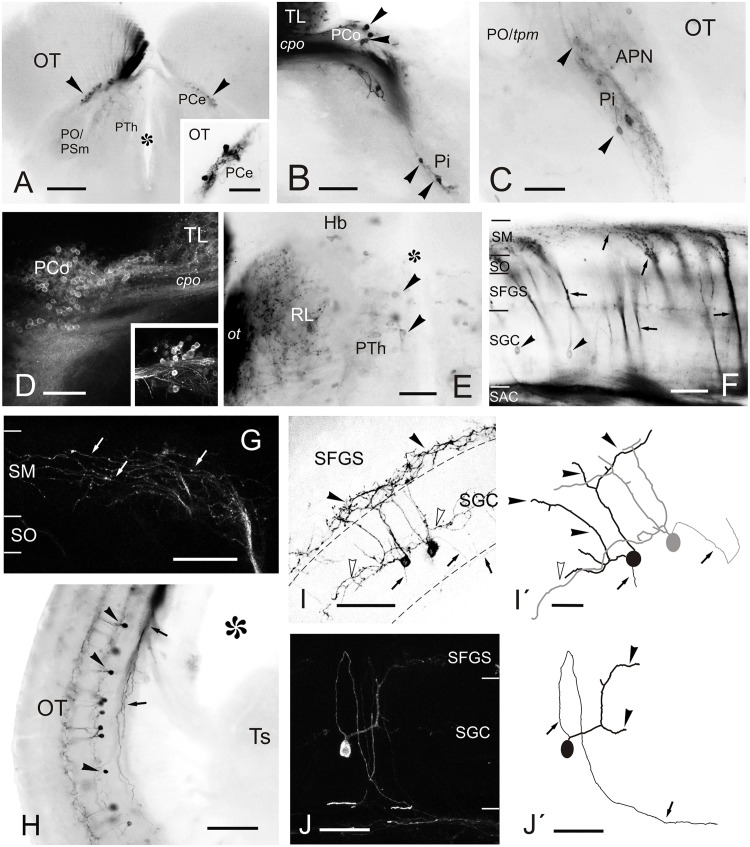
Labeled cells and fibers after direct DiI application to TL. **(A–J)** Photomicrographs of labeled structures observed in transverse sections at diencephalic and mesencephalic levels after DiI application to the TL. **(A)** Labeled cells in the central pretectal nucleus (PCe; arrowheads). **Inset:** detail of labeled cells in the PCe. **(B**,**C)** Retrogradely labeled cells (arrowheads) in the intercalated (Pi; in **B** and **C**) and paracommissural (PCo; in **B**) pretectal nuclei. **(D)** Confocal image showing retrogradely labeled cells in the ipsilateral PCo. Inset: contralateral PCo. **(E)** Faintly labeled cells (arrowheads) in the prethalamus and labeled terminals and fibers in the nucleus rostrolateralis and optic tract. **(F)** Retrogradely labeled tectal cells (black arrowheads) and anterogradely labeled bundles of marginal axons (arrows) through the optic tectum to reach the marginal layer (SM) where they spread giving beaded axon profiles. **(G)** Confocal image of the marginal layer showing a detail of beaded parallel fibers (arrows) spreading from one torofugal tract. **(H)** Retrogradely labeled cells (black arrowheads) and fibers (arrows) in the tectum. **(I)** Inverted image of the projection of a confocal stack with two fluorescent toropetal tectal cells showing dendritic arborization in the SGC (open arrowheads) and ventral SFGS (black arrowheads) and, their axon directed ventrally to the SAC (arrows). **(I’)** Tracing of the cells shown in **(I)**. **(J)** Confocal image of a toropetal tectal cell. **(J’)** Tracing of cell shown in **(J)**. Asterisk, ventricle. For abbreviations, see list. Scale bars: 200 μm **(A)**; 100 μm **(B,H)**; 50 μm **(D,G,I–J,J’)**; 20 μm (inset in **A,C,E,F,I**’).

In the pretectum, retrograde labeled cell bodies were bilaterally observed in the central (PCe), intercalated (Pi), and paracommissural (PCo) pretectal nuclei (Figures 5B,C, 6A–D). In experiments where DiI had diffused from TL into the optic tectum, we observed (in 4 out of 6 experiments) faintly labeled cell bodies in the lateral tier of the prethalamus (formerly the ventromedial nucleus of the thalamus or thalamic eminence) (Figures 5A, 6E). In one of these experiments, we also observed some labeled fibers between the optic tract and the ventromedial thalamic nucleus, in an area that likely corresponds to the nucleus rostrolateralis (Figures 5A, 6E; Saidel and Butler, 1997; Butler and Saidel, 2003; Saidel, 2013).

In the optic tectum, we observed labeled neurons (toropetal), as well as dense bundles of labeled fibers reaching the marginal layer of the optic tectum (Figures 5C–E, 6F–J’). The labeled toropetal neurons were located in the stratum griseum centrale (SGC) (Figures 6F–J’) and showed an average diameter of 10.09μm ± 1.7 SD (*n* = 15). Most cells showed round somatas (*n* = 10/15), although a few piriform ones were also observed (*n* = 5/15). Toropetal neurons have dendrites that branch in two strata (Figures 6I–J’). Their lateral dendrites branch in the external margin of the stratum griseum centrale (SGC) (Figures 6I–J’). Their ascending radial dendrite reaches the deepest layer of the stratum fibrosum et griseum superficiale (SFGS) where it branches forming a plexus with long beaded processes (Figures 6I–J’). The axons of this tectal neuron travel to the stratum album centrale (SAC) and then run to the TL, sometimes following a looping trajectory (Figures 6G,I–J’). Based on location, cell morphology and connectivity, these toropetal cells seem to correspond with Type X cells described in goldfish by Meek and Schellart (1978) and likely with the small multipolar cells of Vanegas et al. (1974). In addition to labeled neurons, labeled fibers were also observed in the optic tectum. Axon bundles labeled from the TL enter the medial optic tectum ventrally through the basal plexiform layer of the stratum album centrale (SAC), ascend in discrete bundles throughout the optic tectum layers and then course and spread in the marginal layer (SM) showing beaded profiles (Figure 6F). This beaded appearance of marginal fibers is suggestive of *en passant* or terminal presynaptic dilatations.

**FIGURE 7:**
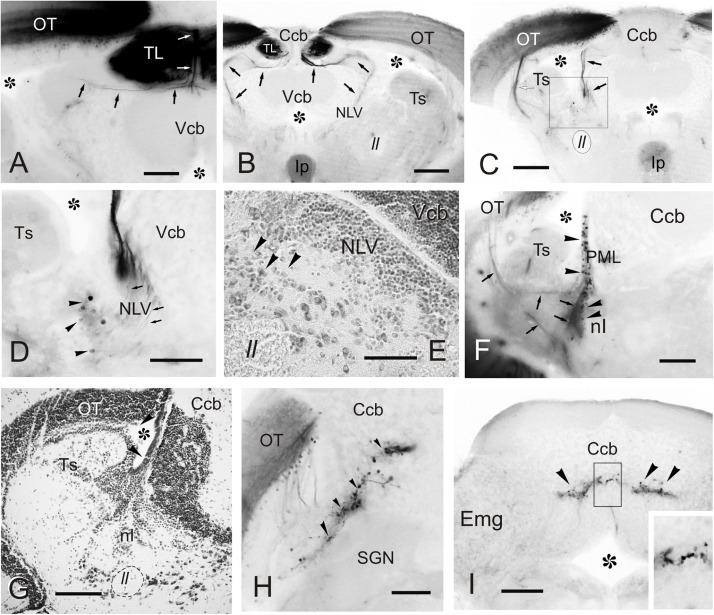
Labeled cells and fibers after direct DiI application to TL. Photomicrographs of transverse sections caudal to the tracer application point showing labeled structures observed after homolateral DiI application to intermediate TL. Ipsilateral side is to the left. **(A–C)** Sections showing the caudal course of the torovalvular tract (arrows) from the labeled TL **(A,B)** that enters and travels bilaterally through the cerebellar valvula. Note in **(C)** the (tecto-)isthmal tract (white arrow) also labeled from some DiI diffusion to the ipsilateral optic tectum. **(D)** Detailed view showing labeled cells ventrolaterally to the nucleus lateralis valvulae (arrowheads) squared in **(C)**, which could represent the subvalvular nucleus in the zebrafish. Note a few labeled fibers from the torovalvular tract traversing the nucleus lateralis valvulae (arrows). **(E)** Nissl stained section showing larger cell bodies (putative subvalvular nucleus, arrowheads) ventrolaterally to the nucleus lateralis valvulae. **(F)** Labeled cells (arrowheads) and fibers (arrows) in the posterior mesencephalic lamina and the nucleus isthmi. **(G)** Nissl stained section showing the posterior mesencephalic lamina (arrowheads). **(H)** Section showing labeled cells and fibers in the optic tectum, as well as cells in the cerebellar body (arrowheads). **(I)** Labeled cells in the granule cell layer of the cerebellar body (arrowheads), detailed in the inset. Asterisk: ventricle. For abbreviations, see list. Scale bars: 200 μm **(B,C,F–I)**; 100 μm **(A,D,E)**.

In the hindbrain, we observed a small fiber bundle that enters the cerebellar valvula medially (hereafter referred to as “torovalvular tract”) and travels along its lateral domains (Figures 7A–C). The tract could be followed through the nucleus lateralis valvulae, where many axons split (Figures 7C,D). At this level, we observed labeled somas located ventrolaterally to the nucleus lateralis valvulae (Figure 7D), which could be the origin of the torovalvular tract. Although these retrogradely labeled cell bodies are very close to the nucleus lateralis valvulae, based on their location and connectivity, they could correspond to the subvalvular nucleus reported in other teleosts (Ito et al., 2003; Xue et al., 2003; Folgueira et al., 2007). In Nissl stained sections of the zebrafish, these cells are larger than those of the nucleus lateralis valvulae (Figure 7E). In addition to the results reported above, we occasionally observed labeled cells in the isthmic nucleus and the torus semicircularis (Figure 7F), together with the isthmo-tectal fiber bundles running along the optic tectum (Figures 7C–G) but we believe these results are due to unwanted DiI diffusion from TL into the optic tectum and/or the intertectal commissure (see section “Connections of the Torus Longitudinalis: Reciprocal DiI Experiments” below), rather than genuine connections to TL. We also observed occasionally labeled cells in the posterior mesencephalic lamina (PML), which connects the tectum and the cerebellum (Grandel et al., 2006; Galant et al., 2016) (Figures 7F,G). Finally, in one experiment where DiI was applied into intermediate TL, we also observed some labeled cells in the granular layer of the corpus cerebelli (Figures 7H,I). In this experiment, DiI did not seem to have diffused into either the valvula or the corpus cerebelli.

### Connections of the Torus Longitudinalis: Reciprocal DiI Experiments

**FIGURE 8:**
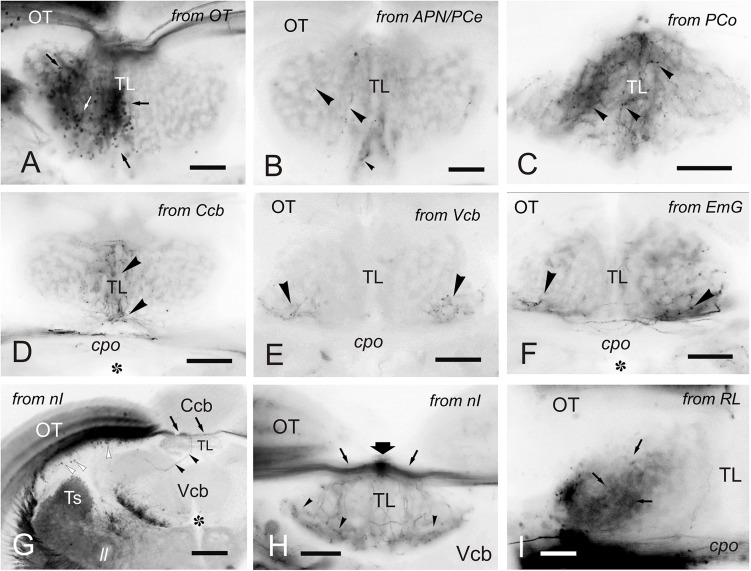
Labeled cells and fibers in TL after DiI application to various regions and nuclei. **(A–I)**, Labeled structures in the TL after unilateral DiI application to the optic tectum **(A)** pretectal nuclei **(B,C)**, cerebellum **(D-F)**, nucleus isthmi **(G,H)** and nucleus rostrolateralis **(I)**. **(A)** Small labeled cells in the ipsilateral TL (arrows) after tracer application to the OT. **(B)** Very few labeled fibers (arrowheads) in the medial TL after DiI application to the APN and PCe region. **(C)** Labeled fibers (arrowheads) in the medial TL after tracer application to the paracommissural nucleus. **(D–F)** Bilaterally labeled fibers (arrowheads) in the ventrolateral TL after unilateral tracer application to the cerebellar corpus **(D)**, cerebellar valvula **(E)**, and granular eminence **(F)**. **(G,H)** Labeled fibers (arrowheads) after unilateral DiI application to the nucleus isthmi region. Note labeled cells in the optic tectum (white arrowheads) and labeled fibers coursing the intertectal commissure dorsal to TL (thin arrows) in **(G)** and **(H)**, so as a dorsal longitudinal fascicle (thick arrow) in **(H)**. Note also some fibers in the ipsilateral torovalvular tract (arrowheads), likely labeled by DiI diffusion to nucleus isthmi neighboring areas (see Section “DISCUSSION”), and bilateral labeled fibers in the TL **(H)**. **(I)**, Ipsilateral faintly labeled cells (arrows) in the TL from the rostrolateral nucleus region Asterisk: ventricle. For abbreviations, see the list. Scale bars: Scale bars: 200 μm **(G)**; 100 μm **(A-F,H)**; 20 μm **(I)**.

A number of reciprocal tracing experiments were done to corroborate and expand the results obtained after tracer application in the TL. Experiments where the tracer was applied to one lobe of OT lead to labeling of a number of small and round granule-like cells homogenously distributed in the ipsilateral TL (Figure 8A). After DiI application to the accessory and central pretectal nucleus, we observed very few labeled fibers bilaterally in TL (Figure 8B). Finally, after DiI application to the pretectal paracommissural nucleus we observed many labeled beaded fibers in TL (Figure 8C).

Tracer application to the different parts of the cerebellum (corpus, valvula, and granular eminence) led to labeled fibers entering the rostral TL through the posterior commissure (Figures 8D–F). Some of these fibers could be originating from the band of granule-like cells observed in the cerebellar corpus after DiI application to intermediate TL (see section “Connections of the Adult Zebrafish TL Visualized by Direct DiI Application”). However, for these experiments involving the cerebellum, it cannot be ruled out that DiI application affected the pretecto-cerebellar tract (Yáñez et al., 2018), which carries projections from pretectal nuclei to the cerebellum. If that were the case, this would imply that pretectal nuclei (PCe, Pi) send axon collaterals to TL and cerebellum. This would also explain why tracer application into TL does not result in labeled cells in the valvula cerebelli and granular eminences.

In a few experiments in which DiI was applied to TL and where the optic tectum was also affected, likely by tracer uptake from the intertectal commissure, we observed a few labeled cells in the nucleus isthmi (section “Connections of the Adult Zebrafish TL Visualized by Direct DiI Application”). This is a visually-related nucleus that maintains bidirectional connections with the optic tectum (Henriques et al., 2019) and is located at the dorsolateral isthmal tegmental region near the cerebellar, lemniscal and gustatory tracts. DiI application to the nucleus isthmi region led to labeling through the isthmo-tectal tract of cells and fibers in the ipsilateral optic tectum (Figure 8G). Some labeled fibers cross the intertectal commissure to the contralateral tectal lobe (Figure 8G). In addition to the isthmo-tectal system, we also observed some labeled fibers in the torovalvular tract that enter the TL medially and reach a compact longitudinal bundle located in the dorsal midline (Figures 8G,H). Fibers in this longitudinal compact bundle run rostrally and spread into the TL giving beaded axons both at rostral and intermediate levels (Figures 8G,H). We consider that this projection, through the torovalvular tract and the longitudinal bundle in TL, could originate from the subvalvular nucleus that lays in the vicinity of the nucleus isthmi (see “Subvalvular Nucleus Projections to the Torus Longitudinalis” and “Anatomical and Functional Considerations of the OT- TL Circuit: Possible Relationship With Other Circuits” in Discussion).

DiI application into the nucleus rostrolateralis led to faint labeling of some TL cells, mainly ipsilaterally in a ventrolateral region of the rostral TL (Figure 8I). A few labeled fibers were also observed in the TL in these experiments, likely originating from the neighboring areas of the nucleus rostrolateralis, such as central pretectal areas.

### Pyramidal Cell Morphology in *Tg(1.4dlx5a-dlx6a:GFP)*^*ot*1^ Fish

**FIGURE 9:**
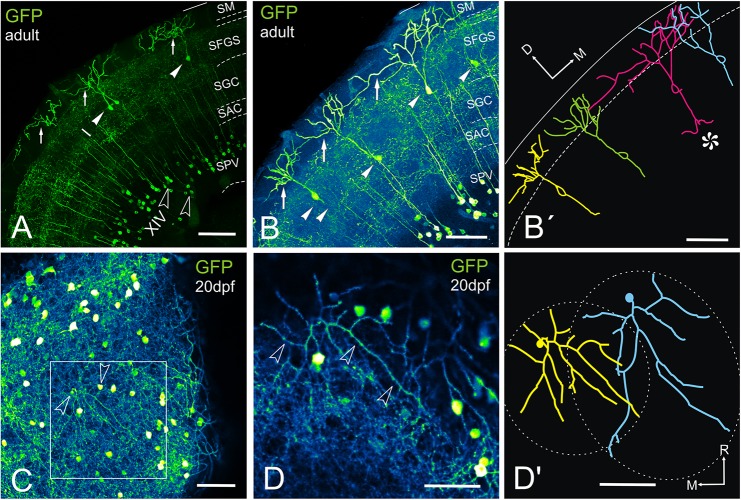
GFP + cells and fibers in the optic tectum of *Tg(1.4dlx5a-dlx6a:GFP)*^*ot*1^. **(A–D’)** Labeled tectal cells (confocal images and tracings) in adult **(A–B’)** and 20dpf **(E–E’)** transgenic zebrafish *Tg(1.4dlx5a-dlx6a:GFP)*^*ot*1^. **(A-D)**, Images projection from confocal stacks. **(A)** Section through the optic tectum of an adult fish showing GFP + pyramidal or type I cells (arrowheads, labeled “I” in the photograph) with their characteristic dendritic arbors (arrows) and cell bodies in the periventricular cell layer that are likely to correspond to type XIV cells from Meek and Schellart (1978) (outlined arrowheads, labeled “XIV” in the photograph). **(B,B’)** Section showing 4 GFP + pyramidal cells **(B)** and their tracing **(B’)**. Note the sparse distribution of the cell bodies (arrowheads). Cell marked with an asterisk in **(B’)** shows quite broad dendritic arbor, overlapping with adjacent ones. Area marked by dotted lines: SM. **(C)** Dorsal view of a whole mount brain of a 20dpf fish showing labeled structures in the optic tectum. Arrowheads point to the cell bodies of the cells traced in **(D’)**. **(D)** Detail of the area squared in **(C)** showing the dendritic arbor (arrowheads) of a pyramidal cell. **(D’)** Tracing of two adjacent pyramidal cells observed in **(D’)**, showing the area covered by their dendritic arbor (dashed line). Note that the dendritic arbor of the cells covers roughly circular and oval areas, and the slight overlap between them. Axis: D, dorsal; M, medial; R, rostral. For abbreviations, see list. Scale bars: 100 μm **(A-C)**; 50 μm **(D,D’)**.

Pyramidal cells (type I cells of Meek and Schellart, 1978) in the optic tectum are the most conspicuous targets of the toro-tectal projection (Laufer and Vanegas, 1974; Vanegas et al., 1974). Serendipitously, we observed that pyramidal cells are GFP positive in *Tg(1.4dlx5a-dlx6a:GFP)*^*ot*1^ juveniles and adults, together with another cell type whose somas are located near the tectal ventricle (Figures 9A,B). Transverse sections of the adult tectum showed clearly the characteristic dendritic arborizations of pyramidal cells in the marginal layer (Figures 9A,B), which cannot be confused with any other tectal type. The other main GFP-positive population labeled in this transgenic line was composed of numerous cells with their perikarya located in the periventricular cell layer (Figures 9A,B). These cells show a slender apical dendrite extending radially through all tectal layers with exception of the marginal zone and give rise to beaded collateral dendrites that branch in three broad neuropil layers (Figure 9A). These periventricular cells correspond with type XIV cells described in goldfish (Meek and Schellart, 1978).

**FIGURE 10:**
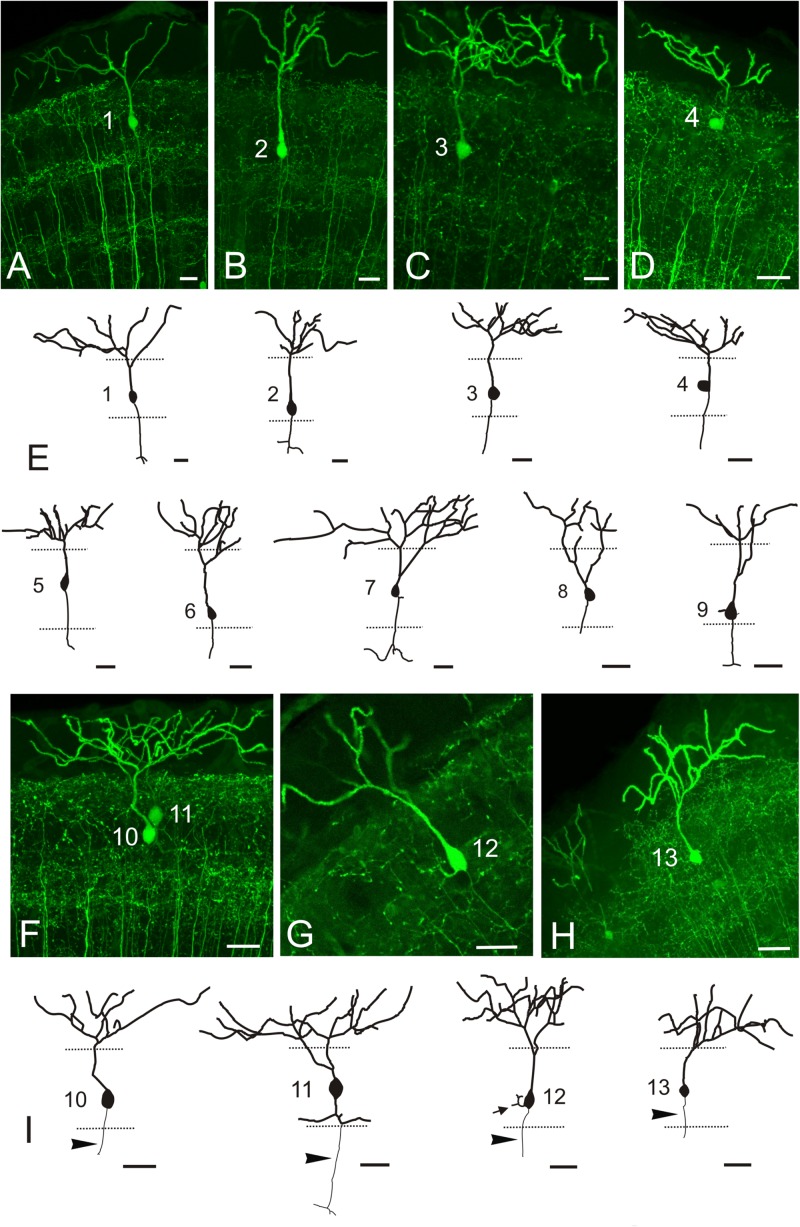
GFP + pyramidal cells in *Tg(1.4dlx5a-dlx6a:GFP)*^*ot*1^. **(A–D,F–G)** Images showing GFP + pyramidal cells in *Tg(1.4dlx5a-dlx6a:GFP)*^*ot*1^ adult fish. All images are projections from confocal stacks. **(E**,**I)** Tracings of 13 pyramidal cells. Apical dendrites are represented with a thicker line than basal ones. Arrowheads in **(I)** point to axon. All cells are numbered 1-13. Scale bars: 20 μm.

In transverse sections, we observed that GFP-positive pyramidal cells are scarce, with their somas more or less regularly spaced and located at different levels of the stratum fibrosum et griseum superficiale (SFGS) (Figures 9A–B’). To determine the extension of the whole dendritic arbor of the pyramidal cells in the marginal layer, we imaged a whole-mount brain of a 20 days post-fertilization fish from a dorsal approach (Figure 9C). At this stage, the marginal layer is about 10% of adult thickness (15 μm thick at 20 dpf *vs*. 150-180 μm thick in the adult). After tracing two pyramidal cells (Figures 9C–D’), we observed that their somas are eccentrically located and their dendritic arbors extend over round and oval areas respectively (Figure 9D’), with little overlap (Figure 9D’). In adult fish, all single-cell morphological analyses were performed in 50-100 μm transverse sections of the optic tectum, so it is likely that parts of the dendritic arbor are missing in the *Z*-axis. For pyramidal cells analyzed in the adult, somas are either pear-shaped (9/13) or round (4/13) (Figures 10A–I and Supplementary Movies 1, 2), with an average width of 8.36 μm ± 1.54 SD. All pyramidal cells studied show a thick apical dendrite (average width 2.02 μm ± 0.67 SD) that branches in the marginal layer (Figures 10A–I), forming a sparse tree of thick, spiny branches that extend laterally in a wide area (average width 117.12 μm ± 40.02 SD). The apical dendrite normally branches away from the cell body, in the proximity of the marginal layer. Only occasionally, the first branching of the apical dendrite occurs quite close to the cell body (cells 7, 8, 11 Figures 10E,I). The lateral extension of apical dendritic arbors varies among neurons, occasionally overlapping with dendritic arbors of adjacent pyramidal neurons. For some cells (9/13), we observed a thinner basal dendrite (1.18 μm ± 0.37) that exits from the basal cell pole (Figures 10A–E) and could be followed to the stratum griseum centrale (SGC). Because there are a number of GFP positive fibers in the strata below the marginal layer, we could not trace the whole extension of the basal dendrite even in high-resolution confocal images. In other teleosts it has been described that an axon originates from the basal dendrite (Vanegas et al., 1974; Xue et al., 2003; Folgueira et al., 2007). In our case, only in one instance did we observe a thinner cell process or neurite, possibly an axon, originating from one of the lateral branches of the basal dendrite (Figure 10I cell 11). In three other cells (Figure 10I cells 10, 12, 13), we could observe a thin basal neurite, likely an axon, originating directly from the soma. Finally, in two cells, we observed a lateral dendrite that seems to branch near the cell body (Figure 10E cell 9, Figure 10I cell 12, and Supplementary Movie 2).

**FIGURE 11:**
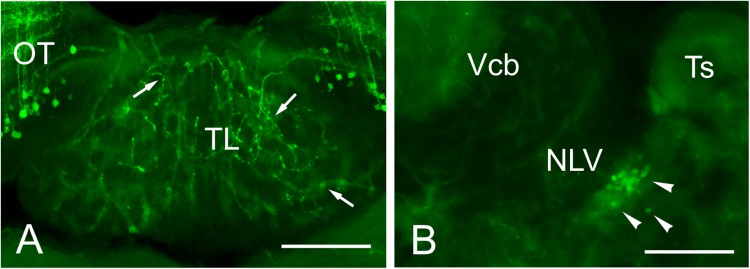
GFP + cells and fibers in *Tg(1.4dlx5a-dlx6a:GFP)*^*ot*1.^ **(A)** GFP positive fibers in TL in *Tg(1.4dlx5a-dlx6a:GFP)*^*ot*1^ adult fish. **(B)** GFP + cell bodies in the putative subvalvular nucleus of a *Tg(1.4dlx5a-dlx6a:GFP)*^*ot*1^ adult fish. Scale bars: 100 μm.

In addition to the somas and fibers labeled in the tectum, we observed a few beaded GFP + fibers entering TL just below the intertectal commissure (Figure 11A). These fibers can be observed from rostral to caudal levels of TL. Among all GFP + cell populations labeled in *Tg(1.4dlx5a-dlx6a:GFP)*^*ot*1^, the best candidate for the origin of these GFP + fibers is the subvalvular nucleus (Figure 11B), which was labeled after experiments involving DiI application into TL (see sections “Connections of the Adult Zebrafish TL Visualized by Direct DiI Application” and “Connections of the Torus Longitudinalis: Reciprocal DiI Experiments” above).

## Discussion

### Structural and Ultrastructural Studies of the Torus Longitudinalis

Three main neuron types were described here in the TL of adult zebrafish based on nucleus size, chromatin condensation and cytoplasm amount surrounding the nucleus. In neurons, it has been shown that the nucleus size and condensation is closely related to cell size and extent of processes, but also to activity within the same cell class (Huber and Gerace, 2007; Skinner and Johnson, 2017). Differences in the nucleus and chromatin condensation in these cells could be either caused by variations in cell activity or genuine differences between cell types. Because of their abundance and characteristics, the medium-size (**C**_1_) and likely the small (**C**_2_) cell populations probably represent granule cells. In the case of the small neurons (**C**_2_), in light microscopy they seemed to have homogeneously stained nuclei, but transmission electron microscopy (TEM) analysis showed differences in chromatin condensation (**Nu**_2_ and **Nu**_2__’_) that must be truly related to differences in cell activity.

Studies in other teleosts have also described different neuron populations in the TL based on cell morphology or functional properties. Early studies in the carp TL showed three types of toral neurons: small (4-5 μm), medium-sized (5-8 μm) and large (10-12 μm) (Ito, 1971). In this study, it is suggested that both small and medium-sized cells belong to the granule cell population, with medium-sized cells differentiating from small cells (Ito, 1971). Rooting from this idea, we wonder if that could be the case also in zebrafish. In this species, proliferating cells have been described in the adult along the medial and dorsal areas of the TL (Zupanc et al., 2005), as well as the ventricular surface (Grandel et al., 2006). TL is also heterogeneous functionally. Electrophysiological recordings showed that in the percomorphs *Holocentrus* and *Eugerres*, and in the goldfish, some TL units respond in a sustained fashion to dimming and others burst with saccadic eye movements (Northmore et al., 1983; Northmore, 1984). Moreover, these responses types are segregated into dorsal/medial and ventral/lateral regions, respectively (Xue et al., 2003; Northmore, 2011). Unlike those species, we observed no clear regional differentiation in zebrafish TL by cell size or connectivity.

We observed that GABAergic (GAD67-positive) neurons represent a small population located mainly in dorsal regions of zebrafish TL, but also in ventrolateral regions. These cells seem to correspond to the larger cells with paler nuclei observed in semithin and ultrathin sections. In another study of the adult zebrafish brain, GAD67 mRNA-expressing cells are observed in TL (see Figure 3 from Mueller and Guo, 2009). This population could be a common feature in the TL of ray-finned fish, as GABAergic cells are also distinguished in dorsal areas of trout TL and are likely the source of the rich GABAergic innervation in the TL neuropil (Folgueira et al., 2007). It seems that this could also be the case in zebrafish, as it also showed rich GABAergic innervation in the TL. Despite the presence of GABAergic neurons in the TL, the majority is probably glutamatergic. The glutamate transporter 1 (vglut1) mRNA is strongly expressed in zebrafish TL (Bae et al., 2009) and a cichlid fish (Maruska et al., 2017), and clearly these cells outnumber the GAD-positive ones in both species. Similarly, most TL neurons are glutamatergic in goldfish (Kageyama and Meyer, 1989).

A number of neuronal processes were observed in the TL with transmission electron microscopy (TEM), including dendrites and axons of toral neurons and the axons from extrinsic toropetal neurons. TL neurons appeared to be the origin of the small compact bundles of unmyelinated thin axons distributed within and between the granule cell clusters. These bundles resemble the parallel fiber bundles ascending through the cerebellar granular layer of mammals toward the molecular layer (see Palay and Chan-Palay, 1974). Tracing experiments in zebrafish revealed that toral cell axons exit the TL, then reach the stratum album centrale (SAC) of the optic tectum and finally ascend in separate bundles to the marginal layer, where they run parallel to the brain surface and form terminals. In addition, TEM observations of the zebrafish TL also revealed many axo-dendritic synapses in its neuropil. At least three types of axon terminals were observed, but correspondence between axon terminals in TL and cells of origin could not be determined. The most characteristic synapses in the zebrafish TL showed wavy-shaped active zones, as also seen in carp (Ito, 1971). However, the zebrafish TL lacked the glomeruli formed by large presynaptic terminals surrounded by several terminal dendrites described in carp (Ito, 1971). Occasionally, we also observed a type of presynaptic terminal in the rostral zebrafish TL that contained large dense-core synaptic vesicles, probably containing either catecholamines or peptides. In adult sections from a previous study in zebrafish (Castro et al., 2006), we observed scattered catecholaminergic fibers in the TL (see also fibers in Figures 4, 5 in Kaslin and Panula, 2001), which could support this interpretation of the TEM results. However, no TH immunoreactive population described previously in zebrafish matches with the toropetal centers described here, so the origin of these fibers cannot be clarified. It is also possible that our experimental approach does not reveal minor projections to the TL, in particular the locus coeruleus that is catecholaminergic in teleosts and extends collaterals throughout the brain (Ekström et al., 1986; Ma, 1994, 1997).

### Reciprocal Toro- Tectal Connections

**FIGURE 12:**
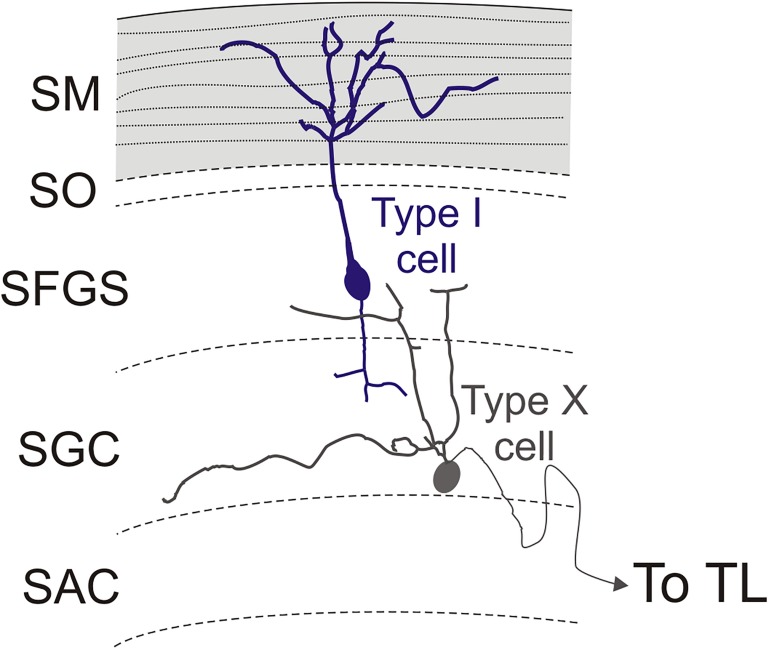
Schematic representation of type I and type X cells in the optic tectum. The stratum marginale is marked in gray. Parallel fibers are represented with dotted lines. Limit between layers in the optic tectum are marked with dashed lines.

We observed a single type of tectal neuron that project to the TL in zebrafish (Figure 12). These neurons showed a clear bi-stratified dendritic lamination in the SGC and the SFGS, which roughly coincides with the morphology of the type X tectal cells described after Golgi staining in the goldfish (Meek and Schellart, 1978). A recent study in zebrafish larva has shown that type X cells are labeled by *id2b* transgene, being able to observe the axon running along TL (DeMarco et al., 2019). In the carp tectum, the toropetal cells reported with retrograde labeling (Ito et al., 2003) appear to be more heterogeneous in appearance and location than those reported here in zebrafish, with cells located in the SFGS, SGC and SAC (Ito et al., 2003; present results). Toropetal cells were also observed in the SGC of holocentrids and salmonids (Xue et al., 2003; Folgueira et al., 2007), although with differences in cell morphology. In holocentrids, somata are bipolar and located midway between the two strata where cell dendrites branch. In trout, most toropetal cells have pear-shaped somata with a conspicuous ascending radial dendrite that branches in a single stratum. Based on cell morphology and the distribution of cell processes in the OT layers, direct synaptic contacts between type X and pyramidal (type I) cells have been suggested (Xue et al., 2003; Northmore, 2017). Our results also showed that direct synaptic contact between these two cell populations is possible (Figure 12) but future studies need to confirm this, maybe by using transsynaptic labeling (Mundell et al., 2015; Beier et al., 2016). In the adult zebrafish tectum, apical and basal dendritic branches of type X neurons extended horizontally in the deepest layer of the SFGS (likely corresponding to sublamina 6 described in the tectum of zebrafish larvae) and in the SGC, respectively. The larval sublamina 6 (SFGS6) receives direct visual inputs from several types of retinal ganglion cells, some of them also sending branches to other arborization fields outside tectum (Robles et al., 2013, 2014). Noticeably, a particular class of retinal ganglion cells shows bistratified axonal projections that reach both the SFGS6 and SGC (Robles et al., 2014). These results match with the distribution of the dendritic fields of type X cells described for the zebrafish adult (present results) and larva (DeMarco et al., 2019). Therefore, it seems that zebrafish type X cells probably receive direct retinal information, among other types of inputs. Results in zebrafish larva have shown afferents to the optic tectum from fifteen different areas (Kunst et al., the dorsal raphe (Yokogawa et al., 2012; Filosa et al., 2016), hypothalamus (Heap et al., 2018a) and thalamus (Heap et al., 2018b), among other areas (Kunst et al., 2019). As a result, this and other areas could modulate the toro-tectal circuit in zebrafish (see “Anatomical and Functional Considerations of the OT- TL Circuit: Possible Relationship With Other Circuits” below).

The efferent projection from TL to the optic tectum (also known as the fasciculi toro-tectales), composed of unmyelinated fibers originating from granule cells, has been recognized in classical studies of the TL (Kudo, 1923; Suzuki, 1965), and demonstrated in zebrafish with the Golgi method (Sajovic and Levinthal, 1982). The main targets of these TL projections are pyramidal cell dendrites in the marginal layer of the ipsilateral optic tectum (Vanegas et al., 1974; Sajovic and Levinthal, 1982). This layer contains beaded marginal (parallel-like) fibers that contact the spiny apical dendrites of pyramidal cells branching in this layer (Vanegas et al., 1974). In this sense, the marginal layer of the optic tectum shows characteristics that resemble the molecular layer of the cerebellum (Bell, 2002; Bell et al., 2008). The zebrafish pyramidal cells were succinctly described with the Golgi method (Sajovic and Levinthal, 1982) and appear to correspond to the type I tectal neurons described in goldfish also with Golgi methods (Meek and Schellart, 1978). Recently, pyramidal cells have been described in zebrafish larva based on the expression of *id2b* transgene (DeMarco et al., 2019). Based on general cell morphology and cell body location (in the SGC and SAC, not in the SFGS), these larval cells do not seem to correspond with the pyramidal (type I) cells reported here. Like TL granule cells, the marginal fiber terminals are glutamatergic in goldfish (Kageyama and Meyer, 1989). In zebrafish, pyramidal cells express the glutamate receptor delta 2 specifically in their apical dendrites (Mikami et al., 2004). GABAergic boutons are also found in the marginal layer of the tectum of zebrafish and trout (Castro et al., 2006; Folgueira et al., 2007), suggesting inputs to this layer from tectal GABAergic neurons. Here we examined pyramidal (type I) neurons in the optic tectum of adult zebrafish using the *Tg(1.4dlx5a-dlx6a:GFP)*^*ot*1^ line, but axon targets of pyramidal cells could not be determined due to the presence of other GFP + fibers in the tectum which obscured the path of the basal processes. Only in a few cases could we see the origin of a thin neurite, probably an axon, either from the cell body or the basal dendrite. In all cases, we were unable to follow the extension of these thin neurites to their terminals. Sajovic and Levinthal (1982) did show that the axon of pyramidal cells arborizes in the SGC and in the SFGS, as described previously in other species (Vanegas et al., 1974; Meek and Schellart, 1978; Xue et al., 2003; Folgueira et al., 2007). In addition to afferents from the TL, pyramidal cells might receive retina afferents (Laufer and Vanegas, 1974; Northmore, 2017). In zebrafish larva, projections from retina ganglion cells reach the SO, SFGS and SGC, as well as a layer between SAC and SPV (Burrill and Easter, 1994, Robles et al., 2013, 2014). So it is possible that pyramidal cells do receive direct retina inputs also in zebrafish. In cyprinids and other teleosts, Golgi staining and other experimental methods (Ramón y Cajal, 1909–1911; Vanegas et al., 1974; Meek and Schellart, 1978; Schroeder et al., 1980; Folgueira et al., 2007) show a bistratified arborization of basal dendrites and a short descending axon in this cell type, which are distributed among the dendrites of the type X neurons projecting to the TL (Meek and Schellart, 1978; Xue et al., 2003; Folgueira et al., 2007). Therefore, these cells appear to participate in a three-neuron TL-OT looping circuit (granule cell-pyramidal/type I cell-type X cell), as previously suggested on the basis of electrophysiological studies (Northmore, 2011).

Regarding the morphology of pyramidal (type I) neurons of the zebrafish tectum, they showed simpler branching of the apical dendrite than that observed in the trout (Folgueira et al., 2007) and much simpler than in holocentrids (Schroeder et al., 1980; Xue et al., 2003). These latter fish exhibit a huge TL and a very thick marginal layer, which correlates with the very long apical dendritic tree of pyramidal neurons (Xue et al., 2003). In *Tg(1.4dlx5a-dlx6a:GFP)*^*ot*1^ zebrafish, pyramidal cells appeared scattered and quite regularly spaced in the OT, at lower density than in other fish (Xue et al., 2004; Folgueira et al., 2007). However, an anatomical study in zebrafish implies a high density of pyramidal cells in the optic tectum based on the expression of parvalbumin 7 (see Figure 11 of Bae et al., 2009). Thus, *Tg(1.4dlx5a-dlx6a:GFP)*^*ot*1^ probably only labels a subset of the pyramidal population in zebrafish. Within this GFP-labeled population, our results revealed a certain degree of morphological variability. Interestingly, a few cells showed a lateral dendrite branching in the SFGS, near the cell body, that is also observed in goldfish (Meek and Schellart, 1978).

### Pretecto-Toral Connections

Results of these tracing experiments and a former study in zebrafish (Yáñez et al., 2018) confirm that the TL receives neural inputs from three visual- and cerebellar-related pretectal nuclei, namely the PCe, the Pi and the PCo. Previous results have revealed that these three nuclei receive retinofugal fibers, project to the cerebellum and, in the case of the PCo, receives projections from the cerebellar corpus (Yáñez et al., 2018). Present experiments of reciprocal labeling from the pretectal nuclei confirmed the presence and bilateral character of these pretecto-toral projections. With little differences in location, three similar pretectal nuclei also project to the TL in the rainbow trout (Folgueira et al., 2008). In this species, like in zebrafish, these toropetal pretectal nuclei also receive retinal projections and project to the cerebellum (Folgueira et al., 2007, 2008). Results in zebrafish are also rather similar to those reported in carp by Ito et al. (2003) who found toropetal cells in the “area pretectalis” and the “paracommissural nucleus.” This pattern is in contrast with that reported in holocentrids, a group of teleosts with large TL and OT marginal layer, in which the PCo, which innervates the cerebellum, was the only pretectal nucleus reported to project to the TL (Xue et al., 2003, 2004). In all the teleosts studied, at least the PCo and likely the PCe relays telencephalic input to the TL (Wullimann and Meyer, 1993; Xue et al., 2003; Folgueira et al., 2004; Yáñez et al., 2018). Taken together, these results indicate that the pretecto-toral projections are quite well conserved in the groups of teleosts investigated so far, that is, cyprinids, salmonids, and holocentrids.

We believe that both the faintly labeled cells in the rostral prethalamic nucleus, (formerly eminentia thalami or part of ventral thalamus) and the fibers labeled in the optic tract observed after DiI application to the TL are probably artefactual. Since no retinal projections to the TL were observed, labeling of fibers in the optic tract must result from DiI diffusion into the optic tectum. Likewise, the rostral prethalamic nucleus projects to the optic tectum in teleosts (Xue et al., 2003; Folgueira et al., 2007) and it is likely that somata are labeled in this nucleus by diffusion from the injection site into the optic tectum. The same argument could be applied to the rostral pretectal nuclei (PCe, Pi) labeled in our experiments. However, these projections were considered genuine because reciprocal experiments showed that pretectal nuclei send projections to the TL through the posterior commissure, not through the optic tectum. In addition, terminal fibers were present in TL after reciprocal tracing experiments in both PCe and Pi (present results) and, in the case of PCe, no tectal projections were described in zebrafish (Yáñez et al., 2018).

### Nucleus Rostrolateralis and the Torus Longitudinalis

The nucleus rostrolateralis (RL) was described as a visually related diencephalic nucleus sporadically distributed in actinopterygians (Butler and Saidel, 1991, 1992, 2003; Saidel, 2013) including zebrafish (Rupp et al., 1996; Wullimann et al., 1996). In the butterfly fish *Pantodon*, this nucleus is reciprocally connected with the TL (Saidel and Butler, 1997). In zebrafish, the RL receives both contralateral retinal projections (Saidel and Butler, 1997; unpublished observations) and seems to send fibers bilaterally to the TL (present study). The zebrafish RL is also considered as a nucleus afferent to the habenula (Turner et al., 2016), but it does not seem to project to the interpeduncular nucleus (unpublished observations) unlike in *Pantodon* (Saidel, 2013). A nucleus located in the subhabenular region/eminentia thalami that responds to light and projects to the habenula has been described in zebrafish larvae (Cheng et al., 2017; Zhang et al., 2017). This nucleus might correspond with the nucleus rostrolateralis described in adults (Turner et al., 2016; present results). Most amniotes show direct retinal projections to the ventrolateral and intermediate thalamic nuclei via the medial optic tract (Vanegas and Ito, 1983; Northcutt, 1995). Whether these nuclei correspond with the adult zebrafish RL or to other prethalamic nuclei needs to be further investigated.

### Subvalvular Nucleus Projections to the Torus Longitudinalis

After DiI application into the TL, we observed a group of labeled cells located ventrolaterally to the nucleus lateralis valvulae. These cells are larger than the ones in the nucleus lateralis valvulae and, based on literature, we consider that they probably represent the zebrafish subvalvular nucleus. This projection from the subvalvular nucleus to TL seems to be very conserved in ray-finned fish, as it is present in carp, trout, holocentrids and maybe in *Pantodon buchholzi* (Ito and Yoshimoto, 1990; Wullimann and Roth, 1994; Ito et al., 2003; Xue et al., 2003; Folgueira et al., 2007). This nucleus could be the source of afferents to the TL driving the saccadic bursts (Northmore, 2017). However, the functional significance of this projection is difficult to infer, as there is no data about afferences and other efferences from the subvalvular nucleus.

### Do Toropetal Cerebellar Connections Exist in Zebrafish?

The presence of inputs to the TL from cerebellum or cerebellum-related areas has been described in several teleosts using experimental procedures (from the eminentia granularis: Ito et al., 2003; from the cerebellar corpus: Murakami and Morita, 1987; Wullimann and Northcutt, 1988; Ikenaga et al., 2002; Xue et al., 2003; Folgueira et al., 2006; from the cerebellar valvula, Folgueira et al., 2006; and from the precerebellar nuclei such as the nucleus lateralis valvulae: Ito and Kishida, 1978; Ito and Yoshimoto, 1990). However, based on our results, the existence of reciprocal connections between the TL and cerebellar structures in zebrafish needs to be critically considered, especially given the possibility of unintended labeling of nuclei or neural pathways passing near the point of tracer application, such as the cerebellar tract, the nucleus lateralis valvulae or the valvula itself.

The existence of a tract coursing between the TL and the cerebellar valvula has been described anatomically in perciforms (Muñoz-Cueto et al., 1998) and is consistent with the abolition of saccadic bursting in the TL of goldfish by lesions to the valvula (Northmore, 1984). This tract was also described with classical neurofibrillary methods in studies of various teleosts (Kudo, 1923; Suzuki, 1965). Our results confirmed the existence of a similar fiber tract in zebrafish (torovalvular tract). The origin of this tract could not be assessed, but candidates are the subvalvular nucleus and/or caudal granule cells in the cerebellum.

### Anatomical and Functional Considerations of the OT- TL Circuit: Possible Relationship With Other Circuits

The teleost optic tectum is a complex multilayered structure that receives a number of extra-tectal afferents. The sources and layers of termination of these tectal afferents, retinal and non-retinal, have been experimentally studied in adult goldfish (Grover and Sharma, 1981). At least fifteen morphological neuron types have been recognized with Golgi methods in the optic tectum of adult cyprinids and other teleosts (Ramón y Cajal, 1909–1911; Vanegas et al., 1974; Meek and Schellart, 1978; Vanegas and Ito, 1983), mainly attending to cell size and dendritic lamination patterns. As most of these neuron types extend radial processes branching in various tectal sublayers, they may receive varied afferent inputs from extra-tectal sources (Grover and Sharma, 1981). These neuron types are probably also functionally diverse within each class. For instance, type XIV cells, the most numerous neuron type in the tectum with dendrites that extend radially throughout all tectal layers except the marginal layer (Meek and Schellart, 1978), in zebrafish includes different subtypes based on the expression of different neurotransmitters (GABAergic, cholinergic, etc., Clemente et al., 2004; [Bibr B61][Bibr B13]). Some tectal neurons also participate in tectal circuits via intratectal axon collaterals, as early shown with Golgi methods in teleosts by Ramón y Cajal (1909–1911) and recently in zebrafish larva based on transgene expression (Gebhardt et al., 2019). Moreover, the intricate synaptic architecture of the optic tectum is probably increased by the presence of unconventional contacts between tectal neurons (dendro-dendritic, dendro-somatic, soma-dendritic), as those reported with electron microscopy in the optic tectum of frogs and elasmobranchs (Székely and Lázár, 1976; Manso and Anadón, 1991).

Studies in zebrafish larva report axonal projections from retinal ganglion cells to specific sublayers in the stratum opticum, stratum fibrosum et griseum superficiale, stratum griseum centrale and border between the stratum album centrale and stratum periventriculare (Burrill and Easter, 1994; Robles et al., 2013). This exquisite lamination pattern of retinal afferents implicates that many tectal cell types may synapse with retinal afferents, this including type I and type X cells. In addition to direct retina inputs, visual inputs could also reach the OT-TL circuit through pretectal centers (Yáñez et al., 2018; present results) and the thalamus (Heap et al., 2018b). It has been shown in the zebrafish larva that thalamic inputs reach deep layers of the tectal neuropil, relaying loom information to the tectum (Heap et al., 2018b). A tectal subsystem is also represented by the “optic tectum-nucleus isthmi” circuitry, which has been implicated in selective attentive processes (Northmore and Gallagher, 2003; Northmore, 2011) and maintenance of prey-tracking sequences (Henriques et al., 2019). Our results in zebrafish suggest that there is no direct connection from the nucleus isthmi to the TL. We observed that fibers from the nucleus isthmi travel past the intertectal commissure but do not enter the TL, consistent with results reported in larval zebrafish (Henriques et al., 2019), as well as other fish including carp (Xue et al., 2001; Ito et al., 2003), holocentrids (Xue et al., 2001, 2003) and trout (Folgueira et al., 2007). In larval zebrafish, two types of neurons in the nucleus isthmi have been described, neither projecting to the TL, but projecting to the optic tectum and pretectum (Henriques et al., 2019). In addition to visual and isthmic inputs, zebrafish optic tectum receives afferents from various other origins as shown in other teleosts (Grover and Sharma, 1981). In the larva, the use of transgenic lines has revealed tectal afferents from the hypothalamus (Heap et al., 2018a) and the dorsal raphe (Yokogawa et al., 2012; Filosa et al., 2016). While inhibitory afferents from the hypothalamus may modulate tectal processing (Heap et al., 2018a), inputs from serotoninergic cells in the dorsal raphe seem to affect visual sensitivity (Yokogawa et al., 2012) and behavior depending on feeding state (Filosa et al., 2016). Recently the zebrafish larva has also been used to construct a detailed atlas of brain circuits at single cell level, identifying fifteen afferent areas to the optic tectum (Kunst et al., 2019). Main afferent areas include the previously described thalamus (Heap et al., 2018b) and nucleus isthmi (Henriques et al., 2019), but also the medial octavolateralis nucleus (Kunst et al., 2019). Whilst this study has revealed new wiring principles in the larval tectum, authors indicate that it was very difficult to recognize the TL neuropil and individual layers of the optic tectum (Kunst et al., 2019), probably owing to the small size and late maturation of the TL.

In addition to results in the larva, neurochemical studies in adult zebrafish reveal that the tectum receives fibers containing different regulatory neurotransmitters, such as histamine, dopamine or serotonin (Kaslin et al., 2004). In adult zebrafish, catecholaminergic fibers (TH-ir) were predominantly found in two broad bands: the superficial band (SO, SFGS) and a deeper central band (inner SGC) (Kaslin et al., 2004). Instead noradrenergic, histaminergic, and serotonergic fibers are mainly distributed in the deeper central layers (SFGS and SGC) (Kaslin et al., 2004). In addition, the teleost tectum receives fibers expressing various neuropeptides (isotocin, GnRH, cholecystokinin, galanin, neuropeptide Y, enkephalin, orexin, growth hormone-releasing hormone-like peptide, among others: see Batten et al., 1990; Kaslin et al., 2004; Castro et al., 2009), some of these involved in feeding behaviors. All these substances would modulate tectal functions and thus influence toral activity directly or indirectly.

The TL had been implicated in conveying corollary discharges related to saccadic movements to tectum (Northmore et al., 1983; Northmore, 1984). More recently, the available anatomical and physiological data has been integrated in a model to suggest that TL is involved in the maintenance of selective visual attention during saccades (Northmore, 2017). Visual information comes directly to the optic tectum from the retina and might come indirectly to the TL from visual pretectal nuclei (Robles et al., 2013, 2014; Yáñez et al., 2018). The visuotopically mapped dimming responses of TL most likely derive from the toropetal neurons described here. The Northmore model supposes that this visuotopic dimming information calibrates the saccadic bursts where both converge on the dendritic trees of tectal pyramidal cells (Northmore, 2017). The model is also consistent with the idea that the various cerebellar-like systems employ corollary discharges to compensate for the effects of self-produced sensory stimulation (Bell, 2002; Bell et al., 2008).

Whether the zebrafish TL displays a saccadic response and the afferent source of eye movement information is elusive. Neurons controlling eye position and eye velocity reside in separate brainstem nuclei in teleosts (Pastor et al., 1994). Velocity inputs to premotor neurons in the caudal hindbrain of goldfish are temporally integrated to control eye position during fixations and movement phases (Aksay et al., 2000, 2003). Neurogenetic methods in zebrafish allowed the characterization of neurons in a small area in the hindbrain that generate saccadic movements (Schoonheim et al., 2010). Recently, zebrafish hindbrain integrator neurons have been studied with calcium imaging and electron microscopy (Daie et al., 2015; Vishwanathan et al., 2017). In our experiments in zebrafish, we did not find any similar hindbrain population projecting to the TL. Nor did we find toropetal cells in the region of the oculomotor nucleus, which Wullimann and Roth (1994) found in *Pantodon* and hypothesized may represent an efference copy to the torus longitudinalis during eye saccades occurring in all teleosts. We cannot discern if these negative results are real or caused by the DiI technique used to trace connections. If real, this would indicate that indirect pathways would mediate the TL responses during saccades in cyprinids, including zebrafish. Future studies, maybe using more precise optogenetic tools, are needed to determine with certainty the origin of the saccadic response in TL.

## Data Availability Statement

The raw data supporting the conclusions of this article will be made available by the authors, without undue reservation, to any qualified researcher.

## Ethics Statement

The animal study was reviewed and approved by UCL Animal Welfare Ethical Review Body and the United Kingdom Home Office under the Animal (Scientific Procedures) Act 1986.

## Author Contributions

MF and JY with contributions from IB conceived and designed the study. JY, SR-M, NF-G, MF, and AC acquired the data. MF, JY, SR-M, and RA analyzed and interpreted the data. JY, RA, and MF drafted the manuscript. MF, JY, AC, and IB revised the manuscript.

## Conflict of Interest

The authors declare that the research was conducted in the absence of any commercial or financial relationships that could be construed as a potential conflict of interest.

## Abbreviations

APN: accessory pretectal nucleus
at_1–3_: axon terminal 1-3
ax: axon
ax_m_: myelinated axon
C_1–3_: cell type 1-3
Ccb: cerebellar corpus
cpo: posterior commissure
D: dorsal axis
Den: dendrite
EmG: granular eminence (of the cerebellum)
fr: fasciculus retroflexus
GFAP: glial fibrillary acidic protein
GFP: green fluorescent protein
Hb: habenula
HL: hypothalamic lobes
Ip: interpeduncular nucleus
*itc*: intertectal commissure
ll: lateral lemniscus
M: medial axis
m: mitochondria
nI: nucleus isthmi
NLV: nucleus lateralis valvulae
Nu_1–3_: nucleus type 1-3
och: optic chiasm
OT: optic tectum
ot: optic tract
PCe: central pretectal nucleus
PCo: paracommissural pretectal nucleus
Pi: intercalated pretectal nucleus
PML: posterior mesencephalic lamina
PO: posterior pretectal nucleus
PSm: magnocellular superficial pretectal nucleus
PTh: prethalamic nucleus
R: rostral axis
Rh: rhombencephalon
RL: nucleus rostrolateralis
SAC: stratum album centrale
SFGS: stratum fibrosum et griseum superficiale
SGC: stratrum griseum centrale
SGN: secondary gustatory nucleus
SM: stratum marginale or marginal layer
SPV: stratum periventriculare
SV: subvalvular nucleus
SV2: synaptic vesicles 2 protein
T: telencephalon
TL: torus longitudinalis
tpm: pretecto-mammillary tract
Ts: torus semicircularis
Vcb: cerebellar valvula.
