# Supplementary material for: Anatomy and Connectivity of the Torus Longitudinalis of the Adult Zebrafish
Source: Front Neural Circuits. 2020 Mar 13;14:8. doi: 10.3389/fncir.2020.00008 (PMC7082427; doi:10.3389/fncir.2020.00008)
Supplement: Supplementary file 1 [file Table_1.DOCX]

Supplementary Material

# Supplementary Data

Supplementary Movie 1. Movie through the optical sections that compose a single confocal stack showing two GFP positive type I cells in *Tg(1.4dlx5a-dlx6a:GFP)^ot1^*.

Supplementary Movie 2. Movie through the optical sections that compose a single confocal stack showing a GFP positive type I cell in *Tg(1.4dlx5a-dlx6a:GFP)^ot1^*. Note that lateral dendrite.
